# Setting research priorities to reduce global mortality from preterm birth and low birth weight by 2015

**DOI:** 10.7189/jogh.02-010403

**Published:** 2012-06

**Authors:** Rajiv Bahl, Jose Martines, Nita Bhandari, Zrinka Biloglav, Karen Edmond, Sharad Iyengar, Michael Kramer, Joy E. Lawn, D. S. Manandhar, Rintaro Mori, Kathleen M. Rasmussen, H. P. S. Sachdev, Nalini Singhal, Mark Tomlinson, Cesar Victora, Anthony F. Williams, Kit Yee Chan, Igor Rudan

**Affiliations:** 1 Department of Child and Adolescent Health and Development, World Health Organization, Geneva, Switzerland; 2 Centre for Health Research and Development, Society for Applied Studies, New Delhi, India; 3 Andrija Štampar School of Public Health, School of Medicine, University of Zagreb, Croatia; 4 London School of Hygiene and Tropical Medicine, London, UK; 5 Action Research & Training for Health, Udaipur, India; 6 Departments of Pediatrics and of Epidemiology and Biostatistics, McGill University Faculty of Medicine, Montreal, Canada; 7 Saving Newborn Lives – Save the Children, Cape Town, South Africa.; 8 Mother and Infant Research Activities, Kathmandu, Nepal; 9 Department of Global Health Policy, Graduate School of Medicine, the University of Tokyo, Japan; 10 Division of Nutritional Sciences, Cornell University, Ithaca, NY, USA; 11 Sitaram Bhartia Institute of Science and Research, New Delhi, India; 12 Department of Pediatrics, University of Calgary, Canada; 13 Department of Psychology, Stellenbosch University, Stellenbosch, South Africa; 14 Federal University of Pelotas, Pelotas, Brazil; 15 St George's, University of London, London, UK; 16 Nossal Institute for Global Health, Melbourne University, Melbourne, Australia; 17 Centre for Population Health Sciences, The University of Edinburgh Medical School, Edinburgh, Scotland, UK; * Equal authors’ contributions; † Staff of the World Health Organization

## Abstract

**Aim:**

This paper aims to identify health research priorities that could improve the rate of progress in reducing global neonatal mortality from preterm birth and low birth weight (PB/LBW), as set out in the UN's Millennium Development Goal 4.

**Methods:**

We applied the Child Health and Nutrition Research Initiative (CHNRI) methodology for setting priorities in health research investments. In the process coordinated by the World Health Organization in 2007–2008, 21 researchers with interest in child, maternal and newborn health suggested 82 research ideas that spanned across the broad spectrum of epidemiological research, health policy and systems research, improvement of existing interventions and development of new interventions. The 82 research questions were then assessed for answerability, effectiveness, deliverability, maximum potential for mortality reduction and the effect on equity using the CHNRI method.

**Results:**

The top 10 identified research priorities were dominated by health systems and policy research questions (eg, identification of LBW infants born at home within 24–48 hours of birth for additional care; approaches to improve quality of care of LBW infants in health facilities; identification of barriers to optimal home care practices including care seeking; and approaches to increase the use of antenatal corticosteriods in preterm labor and to improve access to hospital care for LBW infants). These were followed by priorities for improvement of the existing interventions (eg, early initiation of breastfeeding, including feeding mode and techniques for those unable to suckle directly from the breast; improved cord care, such as chlorhexidine application; and alternative methods to Kangaroo Mother Care (KMC) to keep LBW infants warm in community settings). The highest-ranked epidemiological question suggested improving criteria for identifying LBW infants who need to be cared for in a hospital. Among the new interventions, the greatest support was shown for the development of new simple and effective interventions for providing thermal care to LBW infants, if KMC is not acceptable to the mother.

**Conclusion:**

The context for this exercise was set within the MDG4, requiring an urgent and rapid progress in mortality reduction from low birth weight, rather than identifying long-term strategic solutions of the greatest potential. In a short-term context, the health policy and systems research to improve access and coverage by the existing interventions, coupled with further research to improve effectiveness, deliverability and acceptance of existing interventions, and epidemiological research to address the key gaps in knowledge, were all highlighted as research priorities.

The UN's Millennium Development Goal 4 (MDG4) states that childhood mortality should be reduced by two thirds between 1990 and 2015, but assessments show that the progress in mortality reduction has been disappointing in some countries [1,2]. The main reason usually proposed to explain slow progress is insufficient knowledge on how to implement existing cost-effective interventions and achieve greater coverage of these interventions in low-resource settings [3]. Generating this knowledge is a task for health research that should aim to improve efficiency, effectiveness and equity in implementation of child survival interventions in low and middle-income countries. The most recent World Health Report published by the World Health Organization (WHO) in 2012, entitled “No Health without Research”, has also focused on this issue [4,5]. Through this flagship report, the WHO tried to highlight the importance of health research in reducing the burden of disease and disability in the world and “…*to provide new ideas, innovative thinking, and pragmatic advice for member states on how to strengthen their own health research systems*” [5].

To assist policy makers and donors alike in understanding the potential of different research avenues to contribute to reducing the burden of disease and disability, the Child Health and Nutrition Research Initiative (CHNRI) recently developed a methodology that allows systematic listing and transparent scoring of many competing research options, thus exposing their strengths and weaknesses [6-8]. The Department of Maternal, Newborn, Child and Adolescent Health and Development (MNCAHD) of the WHO has used this methodology to identify health research priorities to tackle five major causes of child deaths, which are thought to underlie two-thirds of all child deaths globally [9]. The most recent estimate reported 8.8 million deaths in children younger than 5 years worldwide in the year 2008, and the main causes were pneumonia (18%), diarrhea (15%), preterm birth complications (12%), neonatal infections (10%) and birth asphyxia (9%) [9]. The results of the CHNRI process coordinated by the World Health Organization to identify research priorities to reduce the mortality burden from childhood pneumonia, diarrhea, birth asphyxia and neonatal infections have already been published [10-13].

The cause “preterm birth complications”, which comprises the old causes “preterm birth” and “low birth weight” (PB/LBW), is on a continuous rise as a proportional cause of child deaths globally and it may become the leading cause over the next decade, as the importance of infectious diseases steadily decreases. Currently, PB/LBW are estimated to cause around 1 million deaths each year [9]. Unfortunately, research interest and investments in preventing neonatal deaths from PB/LBW have not been commensurate with the importance of LBW as the leading child killer [14,15]. In this paper, we present the results of the CHNRI process to set research priorities to reduce the mortality burden from PB/LBW within a context and time frame of the UN’s Millennium Development Goal 4.

## METHODS

**Figure 1:**
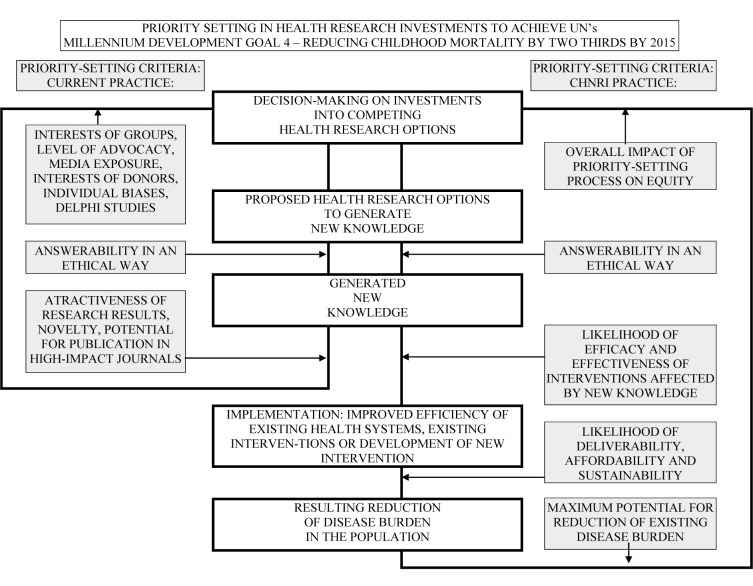
CHNRI’s conceptual framework showing key steps required to get from investments in health research options to decrease in burden of death, disease or disability. The framework identifies criteria to discriminate between likelihoods of success of competing research options: (i) answerability; (ii) effectiveness; (iii) deliverability; (iv) maximum potential for disease burden reduction; and (v) predicted impact on equity in the population (right side). These criteria are not necessarily what drives investment decisions in health research today (left side) [6-8].

The CHNRI methodology for setting priorities in health research investments was proposed as a tool that could be used by those who develop research policy and/or invest in health research [6-8]. This aims to assist policy makers to understand the full spectrum of research investment options and the potential risks and benefits that can result from investments in different research. It also assesses the likelihood of achieving reductions of persisting burden of disease and disability through investments in health research. The CHNRI methodology has 4 stages: (i) input from investors/policy-makers (who define the context and the criteria for priority setting); (ii) input from a larger group of technical experts (who propose, list systematically and then independently score many research ideas); (iii) input from other stakeholders (who agree differential weights for the chosen priority-setting criteria according to wider societal system of values) [6-8;16]; and (iv) computation and discussion of the scores and analysis of the agreement between experts. The conceptual framework for the CHNRI methodology is shown in **Figure 1** and **Table 1**. More detailed explanation has been published elsewhere [6-8;16] and is also available in the Online Supplementary Document(Online Supplementary Document) (table w1).

**Table 1:** CHNRI’s starting framework from which listing of many research options (level of 3-to-5-year research program) and research questions (level of individual research papers) were being proposed by technical experts to systematically organize 82 research ideas

Research instrument	Research avenue	Research option	Research question
Epidemiological research	Measuring the burden	Technical experts were invited to use categorization of research avenues and instruments to systematically propose a number of ‘research options’ within each of the avenues; ‘research options’ correspond to the level of 3-to-5-y research program	Technical experts were invited to propose a number of very specific ‘research questions’, corresponding to the title of individual research papers, within each of the ‘research avenues; eventually, after consolidation and removing of duplicate ideas, 82 such questions were retained for scoring
	Understanding risk factors		
	Evaluating the existing interventions		
Health policy and systems research	Studying capacity to reduce exposure to proven health risks		
	Studying capacity to deliver efficacious interventions		
Research to improve existing interventions	Research to improve deliverability		
	Research to improve affordability		
	Research to improve sustainability		
Research for development of new interventions	Basic research		
	Clinical research		
	Public health research		

### Input from investors/policy makers

The WHO Maternal, Newborn, Child and Adolescent Health and Development program (MNCAHD) coordinated a large international exercise in 2007-2008, involving more than 200 experts from about 80 different countries, to identify health research priorities that could directly tackle the main causes of global child mortality: pneumonia, diarrhea, birth asphyxia, preterm birth/low birth weight and neonatal infections. The aim was to inform key global donors, public investors in health research, and international agencies on research investment policies that could support efforts to accelerate the progress toward the MDG4. Thus, the context for this exercise was a short-term one, set within the MDG4 and requiring an urgent and rapid progress in mortality reduction from childhood pneumonia rather than identifying long-term strategic solutions of the greatest potential. While defining this context, the WHO also recognized the importance of context-specific issues at local or regional levels, the large problem of pneumonia morbidity, and the beneficial effects of investments in the improvement of malnutrition and other cross-cutting and cross-sectoral issues [17,18]. Further details are provided in the Online Supplementary Document(Online Supplementary Document) (table w1).

### Input from technical experts

Individuals with a wide range of technical expertise and regional representation were recruited to participate. A large list of research questions was drafted by the technical expert group based on recent systematic reviews and a survey of experts. Eventually, 21 researchers with interest in child, maternal and newborn health suggested 82 research ideas that spanned across the broad spectrum of epidemiological research, health policy and systems research, improvement of existing interventions and development of new interventions. They were organized using the CHNRI framework for listing research questions, shown in **Table 1**. The expert group then reviewed the questions, refining and reformulating them to allow the scoring. The final questions were sent to each technical group member for scoring. The priority-setting criteria that were adopted were: (i) answerability (in an ethical way); (ii) likelihood of effectiveness; (iii) likelihood of deliverability, affordability, and sustainability; (iv) maximum potential impact on mortality reduction; and (v) predicted impact on equity. The CHNRI framework for scoring research questions is shown in **Table 2** [7,8]. Further details are provided in the Online Supplementary Document(Online Supplementary Document) (table w1).

**Table 2:** Questions answered by technical experts to assign intermediate scores for each criterion to 82 competing research ideas*

*CRITERION 1:* Likelihood that research would lead to new knowledge (enabling a development / planning of an intervention) in an ethical way.
1. Would you say the research question is well framed and endpoints are well defined?
2. Based on: (i) the level of existing research capacity in proposed research; and (ii) the size of the gap from current level of knowledge to the proposed endpoints; would you say that a study can be designed to answer the research question and to reach the proposed endpoints of the research?
3. Do you think that a study needed to answer the proposed research question would obtain ethical approval without major concerns?
***CRITERION 2:* Assessment of likelihood that the intervention resulting from proposed research would be effective.**
1. Based on the best existing evidence and knowledge, would the intervention which would be developed / improved through proposed research be efficacious?
2. Based on the best existing evidence and knowledge, would the intervention which would be developed / improved through proposed research be effective?
3. If the answer to either of the previous two questions is positive, would you say that the evidence upon which these opinions are based is of high quality?
***CRITERION 3:* Assessment of deliverability, affordability and sustainability of the intervention resulting from proposed research.**
1. Taking into account the level of difficulty with intervention delivery from the perspective of the intervention itself (eg, design, standardization, safety), the infrastructure required (eg, human resources, health facilities, communication and transport infrastructure) and users of the intervention (eg, need for change of attitudes or beliefs, supervision, existing demand), would you say that the endpoints of the research would be deliverable within the context of interest?
2. Taking into account the resources available to implement the intervention, would you say that the endpoints of the research would be affordable within the context of interest?
3. Taking into account government capacity and partnership requirements (eg, adequacy of government regulation, monitoring and enforcement; governmental intersectoral coordination, partnership with civil society and external donor agencies; favorable political climate to achieve high coverage), would you say that the endpoints of the research would be sustainable within the context of interest?
***CRITERION 4:* Assessment of maximum potential of disease burden reduction.**
As this dimension is considered “independent” of the others, in order to score competing options fairly, their maximum potential to reduce disease burden should be assessed as potential impact fraction under an ideal scenario, ie, when the exposure to targeted disease risk is decreased to 0% or coverage of proposed intervention is increased to 100% (regardless of how realistic that scenario is at the moment - that aspect will be captured by other dimensions of priority setting process, such as deliverability, affordability and sustainability)
Non-existing interventions†
Maximum potential to reduce disease burden should be computed as “potential impact fraction” for each proposed research avenue, using the equation PIF = [S_(i = 1 to n)_ P_i_ (RR_i_-1)] / [S_(i = 1 to n)_ P_i_ (RR_i_-1) + 1]
where PIF is “potential impact fraction” to reduce disease burden through reducing risk exposure in the population from the present level to 0% or increasing coverage by an existing or new intervention from the present level to 100%; RR is the relative risk given exposure level (less than 1.0 for interventions, greater than 1.0 for risks), P is the population level of distribution of exposure, and n is the maximum exposure level.
Existing interventions‡
Maximum potential to reduce disease burden should be assessed from the results of conducted intervention trials; if no such trials were undertaken, then it should be assessed as for non-existing interventions.
Then, the following questions should be answered:
1. Taking into account the results of conducted intervention trials**, or for the new interventions the proportion of avertable burden under an ideal scenario*, would you say that the successful reaching of research endpoints would have a capacity to remove 5% of disease burden or more?
2. To remove 10% of disease burden or more?
3. To remove 15% of disease burden or more?
***CRITERION 5:* Assessment of the impact of proposed health research on equity.**
1. Does the present distribution of the disease burden affect mainly the underprivileged in the population?
2. Would you say that either (i) mainly the underprivileged, or (ii) all segments of the society equally, would be the most likely to benefit from the results of the proposed research after its implementation?
3. Would you say that the proposed research has the overall potential to improve equity in disease burden distribution in the long term (eg, 10 years)?

*Possible answers: Yes = 1; No = 0; Informed but undecided answer: 0.5; Not sufficiently informed: blank.

†Interventions that are in the pipeline, or could be envisaged as a possibility, but have not been licensed for implementation yet.

‡Interventions that have been licensed for implementation, but may or may not have been evaluated and implemented.

### Solicited input from other societal stakeholders

The five criteria for scoring (answerability, efficacy and effectiveness, deliverability, disease burden reduction and effect on equity) may be perceived to be of varying importance and the value given to each criterion may vary with the perspective of stakeholders. For example, parents who have experienced a pneumonia associated death may rate mortality reduction much higher than a research funder who may value answerability, or a health system planner who may be most concerned with deliverability. Hence, CHNRI undertook an exercise to poll a wide range of stakeholders and to weight the criteria based on values assigned by these stakeholders, as described elsewhere [16]. The weights applied in this exercise are explained in detail in the Online Supplementary Document(Online Supplementary Document) (table w1).

### Computation of the research priority scores and average expert agreement

Completed worksheets were returned to the group coordinator. The overall research priority score (RPS) was computed as the mean of the scores for the five criteria [8], weighted according to the input from the stakeholders [16], according to the formula:


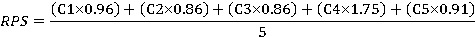


where C designates the scores for relevant criteria.

Average Expert Agreement (AEA) scores were also computed for each research question as the average proportion of scorers that agreed on the 15 questions asked. This is computed for each scored research investment option as:


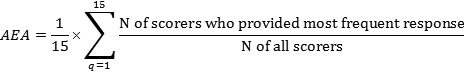


where q is a question that experts are being asked to evaluate competing research investment options, ranging from 1 to 15. For further details regarding the choice of methods, agreement statistics and interpretation see the Online Supplementary Document(Online Supplementary Document) (table w1).

## RESULTS

The scores given to all 82 research questions from individual experts are presented in Online Supplementary Document (table w2), while the final list of priorities with intermediate and final priority scores for all research questions is presented in Online Supplementary Document(Online Supplementary Document) (table w3). In the main body of the paper, **Tables 3** and **4** show the top ten, and also the bottom-ranked ten ideas, respectively, from the 82 proposed and evaluated research questions. The latter three tables transparently present the likelihood for each research question to comply with each of the five chosen priority-setting criteria. Research questions from three broad research domains (health systems and policy research; research to improve the existing interventions; and epidemiological research) feature in the top 10 ranked research questions. The identified research priorities were dominated by health systems and policy research questions (eg, identification of LBW infants born at home within 24-48 hours of birth for additional care; approaches to improve quality of care of LBW infants in health facilities; identification of barriers to optimal home care practices including care seeking; and approaches to increase the use of antenatal corticosteriods in preterm labor and to improve access to hospital care for LBW infants). These were followed by priorities for improvement of the existing interventions (eg, early initiation of breastfeeding, including feeding mode and techniques for those unable to suckle directly from the breast; improved cord care, such as chlorhexidine application; and Kangaroo Mother Care (KMC) and other methods to keep LBW infants warm in community settings). The highest-ranked epidemiological question, ranked 7th, suggested improving criteria for identifying LBW infants who need to be cared for in a hospital.

**Table 3:** Top 10 research questions according to their achieved research priority score (RPS), with average expert agreement (AEA) related to each question

Rank	Proposed research question	Res. type	Answerable?	Effective?	Deliverable?	Burden reduct.?	Equitable?	AEA (%)	RPS (weigh)
1	Identification of low birth weight (LBW) infants within 24-48 h of birth for additional care among those born at home	HPSR	94	89	89	71	89	82.1	84.2
2	Approaches to improve quality of care of LBW infants in health facilities	HPSR	81	100	94	79	72	80.8	83.9
3	Identification of current behaviors, and barriers and supports for optimal home care practices, including care seeking for illness	HPSR	86	78	86	74	97	77.6	82.7
4	Approaches to increase the use of antenatal corticosteriods in preterm labor in resource-poor settings	HPSR	81	91	100	71	81	81.9	82.4
5	Effective interventions for achieving early initiation of breastfeeding including feeding mode and techniques for those unable to suckle directly from the breast	RIEI	86	100	97	67	72	79.0	81.5
6	Approaches to improve access to care for the subset of LBW infants who need hospital care	HPSR	94	82	78	76	81	74.8	81.4
7	Improved criteria for identifying LBW infants who need to be cared for in a hospital	EPI	86	97	81	71	78	75.4	80.8
8	Effectiveness of improved cord care (eg, chlorhexidine application)	RIEI	94	91	81	60	86	78.7	78.8
9	Comparison of Kangaroo Mother Care (KMC) and alternative methods of keeping the LBW infant warm in community settings	RIEI	89	97	78	55	97	82.8	78.6
10	Approaches to increase the use of antibiotics for premature prolonged rupture of membranes in resource-poor settings	HPSR	94	81	75	60	97	75.7	78.2

EPI – epidemiological research, HPSR – health policy and systems research, RIEI – research to improve existing interventions, RDNI – research to develop new interventions

**Table 4:** The bottom 10 research questions according to their overall research priority score (RPS), with average expert agreement (AEA) related to each question

Rank	Proposed research question	Res. Type	Answerable?	Effective?	Deliverable?	Burden reduct.?	Equitable?	AEA (%)	RPS (weigh)
73	Contribution of preterm birth and intrauterine growth retardation to stunting in childhood (increased risk of LBW in next generation of girls subjected to stunting)	EPI	86	39	22	14	81	71.6	43.6
74	Development of safe and effective pharmacological methods of stimulating breastmilk supply	RDNI	64	41	34	33	42	61.8	41.5
75	Approaches to reduce smoking in fathers of unborn chidren during pregnancy	HPSR	67	25	39	21	50	63.2	37.8
76	Development of interventions for activating endogenous surfactant production through gene switching	RDNI	47	54	6	36	39	62.9	36.2
77	Investigating the relationship between sleeping arrangements, infections and SIDS in LBW infants	EPI	56	56	6	26	44	67.6	35.8
78	Determine the degree to which second-hand smoke contributes to LBW among non-smoking women	EPI	64	42	22	10	56	70.3	34.3
79	Development of methods for harmonising the composition of expressed breastmilk to infant requirements without constraining output	RDNI	50	59	13	19	42	67.1	33.9
80	Development of maternal biochemical indicators predicting low birth weight	EPI	69	28	18	26	31	63.3	33.5
81	Investigating the relationship of the home environment and neurocognitive development of LBW infants	EPI	53	50	28	0	58	71.1	31.9
82	Development of interventions for activation of HbA synthesis to ameliorate early anemia in preterm babies	RDNI	53	46	6	21	39	67.2	31.5

EPI – epidemiological research; HPSR – health policy and systems research; RIEI – research to improve existing interventions; RDNI – research to develop new interventions

The predominance of research questions from the domain of health systems and policy research is not surprising, because technical experts were asked to define research priorities that could lead to notable improvements in reduction of PB/LBW mortality by the year 2015. This short time frame benefited research questions that proposed to identify key obstacles to delivery, affordability, and sustainability of implementation of existing cost-effective interventions on a larger scale. The exercise also highlighted the value of investments that aimed to improve and optimise the use of those interventions (alone or in combination) in different contexts, and to develop entirely new approaches that could assist delivery or acceptance of the existing cost-effective interventions.

Research questions seeking to develop new interventions had only three representatives among the 30 highest-ranked questions. This is not surprising given the short specified time frame (the year 2015) by when it would be difficult to envisage new interventions that could have substantial impact, as the CHNRI exercise was conducted in 2007 and 2008. The three ideas that were still encouraged by the experts were: (i) the development of new simple and effective interventions for providing thermal care to LBW infants, if KMC is not acceptable to the mother – which was ranked at the high 12th position on the final list; (ii) identifying micronutrients whose supplementation improves functional outcomes including survival in distinct subgroups of preterm and growth retarded infants; and (iii) development of new simple and effective interventions that prevent infections and improve survival, such as new emollients for massage (see Online Supplementary Document(Online Supplementary Document), table w3).

Among the bottom ranked 10 research ideas, five were questions related to epidemiological research, while further four proposed the development of entirely new interventions and one was health policy and systems research question. The reasons for their low score vary substantially: the ideas on “gene switching to activate endogenous surfactant production” or “harmonising the composition of expressed breastmilk” were neither considered answerable nor equitable. The proposals to “reduce smoking in fathers of unborn children” or “develop maternal biochemical indicators predicting low birth weight” were not considered effective in mortality reduction. Interventions that should be developed from “studying sleeping arrangements, infections and SIDS”, or “activation of HbA synthesis to ameliorate early anemia” were not considered deliverable in the context of low and middle-income countries. In all the cases of 10 research questions with the lowest research priority score, there was a minimal, or entirely non-existent, optimism toward their possible impact on reduction of PB/LBW within the context defined for this exercise.

The CHNRI methodology achieved very good discrimination between the 82 research questions, with the final research priority scores ranging from 84.2 (the highest-ranked research priority) to 31.5 (the lowest-ranked) out of the maximum 100. Furthermore, there was also a substantial gradient in the level of agreement among the scorers on the priority of the 82 questions, investigated by calculating “average expert agreement” (AEA). The AEA scores ranged from 0.533 to 0.828 (with the theoretical minimum of 0.250 and maximum of 1.000). AEA indicates the proportion of scorers that gave the same most frequent answer to an average question they were asked in relation to a specific research investment option. Average expert agreement values are also presented for the top and bottom 10 research questions in **Tables 3** and **4**. Generally, the questions over which the greatest level of overall agreement was observed among the experts were those that also achieved very high overall research priority scores. The greatest point of controversy was the research questions on the role of psychosocial and physical stress (such as manual labor) to preterm birth and intrauterine growth retardation (Online Supplementary Document(Online Supplementary Document), table w3).

## DISCUSSION

Investment in global health research today would benefit from consensus regarding the context, appropriate investment strategies, and co-ordination to achieve significant reduction of the disease burden in the foreseeable future. The present exercise was designed to assist investors and policy makers in making more informed choices on their investments in health research on PB/LBW by making apparent the risks and potential benefits associated with investments in a broad spectrum of health research options. The expected “profit” from investments is associated with generating new knowledge that can be translated into development of new (or improvement of existing) interventions, which are effective, deliverable, affordable, and can reduce the existing burden of disease and disability in an equitable way. The risk is associated with research that is not likely to be answerable, or that develops products unlikely to be effective, deliverable, affordable, or sustainable by those who need them most. Investors' preference for high-risk investment in health research is particularly questionable when it is occurring in a context that requires urgent progress, such as PB/LBW mortality. The focus on complex challenges of implementation (ie, improving health systems, training health workers including poorly educated village health workers, improving drug supply and delivery at community level, etc.), highlighted in this exercise, was reflected in many research questions being ranked near the top of the list of overall priorities.

The context for this exercise was set within the MDG4, requiring an urgent and rapid progress in mortality reduction from low birth weight, rather than identifying long-term strategic solutions of the greatest potential. In a short-term context, the health policy and systems research to improve access and coverage by the existing interventions, coupled with further research to improve effectiveness, deliverability and acceptance of existing interventions, and epidemiological research to address the key gaps in knowledge, were all highlighted as research priorities.

Although the advantages of the CHNRI methodology represent a serious attempt to deal with many issues inherent to a highly complex process of research investment priority setting, there are still concerns over the validity of the CHNRI approach and related biases. One of them is related to the fact that many possible good ideas (“research investment options”) may not have been included in the initial list of research options that was scored by the experts, and to the potential bias toward items that get the greatest press. Another concern over the CHNRI process is that its end product represents a possibly biased opinion of a very limited group of involved people. In theory, a chosen group of experts can have biased views in comparison to any other potential groups of experts. Those limitations are described and discussed in greater detail in the Online Supplementary Document(Online Supplementary Document) (table w1).

The implementation of the CHNRI methodology showed that, within the context of MDG4, a better balance should be achieved between specific domains of health research. Along with continuing strategic long-term investments in new interventions, which represent high-risk high-profit strategies, the CHNRI process suggested that more attention should be given to health policy research, health systems research, operations research, and research that addresses political, economic, social, cultural, behavioral, and infrastructure issues surrounding the problem of child mortality from PB/LBW. These domains of health research are rarely recognized as attractive by investors in health research because their results are unlikely to grab the newspaper headlines, get published in journals with high impact factors, or lead to patents and commercial products. Yet, they can generate new knowledge that can be very helpful in achieving real progress in disease burden reduction. The identified priorities are also in good agreement with the research supported by WHO’s MNCAHD Department at present. They emphasize the evaluation of existing interventions and the development and testing of new delivery approaches for existing interventions. They also highlight the value of research on preventive measures, with research on new interventions being downplayed within the short-term context.

## CONCLUSIONS

The context for this exercise was set within the MDG4, requiring an urgent and rapid progress in mortality reduction from PB/LBW, rather than identifying long-term strategic solutions of the greatest potential. In a short-term context, the health policy and systems research to improve access and coverage by the existing interventions, coupled with research to improve deliverability of existing cost-effective interventions in low resource contexts, and epidemiological research to address the key gaps in knowledge, were all highlighted as research priorities. These questions are mainly targeted at better understanding the barriers toward implementation, effectiveness and optimization of use of available interventions and programmes. If progress toward reduction of global PB/LBW mortality is to be improved by 2015, these are the research questions that are most likely to be of greatest importance. However, very few donors agencies recognize the importance of these domains of health research to readily invest in those options [14,15,18]. The core group of CHNRI experts made several serious attempts to influence the key donors and point to this gap and serious imbalance in health research investing between “upstream” and “downstream” health research and aims to evaluate the results of the CHNRI process conducted by the WHO at the levels of research output from academic institutions, changes in donor investment priorities, and health research policy changes at the main international organizations.
