# Supplementary material for: Setting research priorities to reduce global mortality from preterm birth and low birth weight by 2015
Source: J Glob Health. 2012 Jun;2(1):010403. doi: 10.7189/jogh.02-010403 (PMC3484758; doi:10.7189/jogh.02-010403)
Supplement: Online Supplementary Document [file jogh-02-010403-s001.pdf]

Online supplementary document

Table w1. The CHNRI methodology for setting priorities in health research investments.

STAGE 1: Defining the context and criteria for priority setting<br><br>Specifying the context a priori is a critical part of the CHNRI process, because priority scores for many research investment options may change substantially according to different contexts. The context for this exercise was defined to address research investment priorities that could assist in achieving UN's MDG4. This is a goal on which global consensus has been reached and wide political commitment has been made.<br><br>The context was specified by the WHO Child and Adolescent Health as follows:<br><br>• Burden of disease of interest: deaths from preterm birth and low birth weight (PB/LBW);<br>• Population of interest: children under 5 years of age in all developing countries, where nearly all cases of PB/LBW deaths occur;<br>• Existing policy/target: reduction of PB/LBW mortality by two thirds by 2015 (in order to contribute to the achievement of the UN's MDG4)<br>• Level of urgency: high (because the goal is not being achieved)<br>• Time frame: to achieve detectable improvement in the rate of PB/LBW mortality reduction by 2015 or soon thereafter;
STAGE 2: Choice of technical experts, systematic listing and scoring of research investment options<br><br>The co-ordinators of the project for WHO Child and Adolescent Health (RB and JM) invited a group of 21 international technical experts with interest in PB/LBW research to participate in the CHNRI process. The selection of experts was based primarily on their track record of conducting research of high quality for many years on the topic of PB/LBW in low and middle income countries. Every effort was made to invite a mix of people with different backgrounds (clinicians, epidemiologists, public health experts, program leaders and basic scientists) and from different countries (both developed and developing ones), so that the mix contains a diversity of views from the wider research community. Every expert scored only 2 criteria of his greatest expertise, which led to each research question being assessed independently by 16 different experts who accepted participation in the scoring. This limited the potential impact of any single expert on overall research priority scores.<br><br>The first task of the technical experts was to propose a large spectrum of research questions in a systematic way, according to the CHNRI framework for listing research questions (**Table w2**). The conceptual framework for this process was described in detail elsewhere [7,8]. The co-ordinators from WHO collected all the proposed ideas from each of the experts independently by e-mail. The process was open-ended and it initially yielded 82 research questions from 21 experts. Then the list of research questions was consolidated and worded to make the new knowledge, that was proposed to be generated, apparent to all the scorers. In producing this list, the co-ordinators limited the overlap between proposed ideas and ensured that the research questions were phrased in a way that would make the CHNRI scoring process applicable to each research question. We feel that the final list of 82 questions covers the wide spectrum of all possible questions.<br><br>The second task of the experts was to score all research questions independently, according to the five agreed criteria. For each of the 82 research questions and each criterion, each of the 16 experts who agreed to take part in this step answered three questions targeted to assess the

likelihood of the proposed research to comply with the priority-setting criterion (see **Table 2**). This task was completed by all 16 participating experts, each one choosing 2 criteria closest to his/her expertise. The entire process was conducted and completed via e-mail between October 2007 and June 2008. Further information on methods related to this part of the priority-setting process were presented elsewhere in greater details [7,8].

STAGE 3: Community involvement - input from larger group of stakeholders

CHNRI methodology ensures community involvement through incorporating the opinions and values from a broader group of stakeholders (e.g. expected recipients of the research, taxpayers who fund health research, health workers, journalists and media, experts in ethics, law, political science, etc.) [16]. Stakeholders lack expertise to directly decide research priorities, but their opinions and values can still be incorporated by weighing the chosen priority-setting criteria according to their perceived importance. In three separate exercises that took place between March and June 2006, CHNRI consultants interviewed three different groups of stakeholders [16]. We decided to use weights provided by the group of stakeholders most appropriate to this exercise (members of an international priority setting network co-ordinated from the University of Toronto) to compute the overall priority score for each of the 82 research options. More detailed explanations on the rationale and methods for including stakeholders' opinions in the process are presented elsewhere [16].

STAGE 4: Computation of "research priority scores"

All the experts answered the questions listed in **Table 1** by ‘Yes’ (1 point) or ‘No’ (0 points). They were also allowed to declare an informed but undecided answer (0.5 points) or declare themselves insufficiently informed to answer the question (missing input). Thus, the proposed research questions got a score for each of the five criteria as "the proportion of maximum possible points scored when an answer was given" (i.e., excluding the missing input). They represent a direct measure of collective optimism of the scorers. Each of the 82 listed research questions received five intermediate scores (each ranging between 0-100%), which were then multiplied by 100 and weighted according to the input from the stakeholders. The weights were applied as follows: a weight of 1.75 was given to the criterion "maximum potential for disease burden reduction"; 0.96 to "answerability in an ethical way"; 0.91 to "predicted effect on equity in the population"; 0.89 to "deliverability, affordability and sustainability"; and 0.86 to the criterion "potential contribution to effectiveness" [8,16]. The overall research priority score (RPS) was then computed as the weighted mean of all five intermediate priority scores. The exact scores given to all 82 research questions from individual experts are presented in **supplementary Table w2**. The final list of priorities with intermediate and final priority scores for all 82 proposed research questions is presented in **supplementary Table w3**.

Assessment of agreement between scorers

CHNRI methodology has the ability to expose the issues of the greatest agreement and controversy. This allows more focused discussion among experts following this exercise, and informs the investors and policy makers about the amount of controversy that surrounds each research question. The datasets that CHNRI methodology produces are not appropriate for application of the usual Kappa agreement statistics, which has been discussed in detail elsewhere [8].

For each evaluated research investment option, average expert agreement (AEA) is informing us, for an average question, what proportion of scorers gave the same most frequent answer. This parameter satisfactorily accounts for missing answers, is unaffected by responses of ‘undecided’, and is also unaffected by the varying number of scorers per criterion and differences in scorer composition for the different criteria. In AEA computation, all 4 possible

responses (“Yes”, “No”, “Neither” and “Don’t know”) are treated as a valid response. Therefore, missing values (“Don’t know”) are also treated as a possible response. If the substantial proportion of the experts say that they “Don’t know” the answer, AEA will reflect this and reduce the level of overall agreement, rather than increase it.

Advantages and limitations of the CHNRI methodology

The applied CHNRI methodology proved to be helpful to systematically list and score a very large number of specific research questions, as shown recently in exercises conducted at national level in South Africa, and at global level for mental health research issues, zinc deficiency, childhood pneumonia, childhood diarrhoea, neonatal infections, primary health care, disability groups, etc. (see http://www.chnri.org/publications.php). Other advantages of the CHNRI process include its systematic nature, transparency, well defined (a priori) context and criteria chosen for discriminating between research investment options, a highly structured way in which relevant information is obtained from the scorers, independent scoring that limits influence of strong-minded individuals on the rest of the scorers, its informative and intuitive quantitative outputs and ability to expose points of greatest agreement and controversy.

Still, the methodology is not free of several possible biases. Although the advantages mentioned above represent a serious attempt to deal with many issues inherent to a highly complex process of research investment priority setting, there are still concerns over the validity of the CHNRI approach and related biases. One of them is related to the fact many possible good ideas (“research investment options”) may not have been included in the initial list of research options that was scored by the experts, and to the potential bias towards items that get the greatest press. The spectrum of research investment options listed initially in this exercise was derived through a systematic process, but it is not endless and it cannot ever cover every single research idea. Specific research methodologies (i.e. randomized clinical trials, etc.) are not mentioned because the research questions listed in that exercise are unlikely to be answered by a single well-defined study. Therefore, the CHNRI process aims to achieve reasonable coverage of the spectrum of possible ideas. After the completion of the exercise, approximate scores and ranks for some specific research questions that are missing in the initial systematic list could still be estimated – either by relating them to the most similar questions on the list or by having those missed questions scored by a single expert (or by a group), using the CHNRI framework and then comparing the computed score to all other scores received for the originally listed research options.

Another concern over the CHNRI process is that its end product represents a possibly biased opinion of a very limited group of involved people. In theory, a chosen group of experts can have biased views in comparison to any other potential groups of experts. However, the number of people globally who possess enough experience, expertise and knowledge on the topic (in this case, PB/LBW) to be able to judge a very diverse spectrum of research questions is rather limited (although certainly much larger than the group that we eventually selected). If one thinks of this “global pool of technical experts” as the whole population that could theoretically be used to solicit expert opinion on the questions that need to be asked, we then selected a “sample” from that population, based on their track record in research on PB/LBW. Given that the “sample” of the experts chosen for this exercise was one of the largest and the most diverse to conduct a CHNRI exercise to date, while the number of experts in this neglected health problem globally is not large, we doubt that there would be considerable differences in the composition of the initial list of questions (or results of the scoring process) if some other group of experts had been selected.

Obviously, CHNRI methodology is not free of bias that results from the choice of the experts, and different groups of experts may indeed have quite different opinions. However, the larger and more diverse the group of chosen experts, the less likely is that the results of their scoring

would significantly deviate from the output of any other large and diverse expert group, chosen from a limited “pool of global technical experts on PB/LBW”.

Validation of CHNRI methodology

CHNRI methodology combines two ideas:

(i) “Principal component analysis” - a statistical technique which reduces a very complex system of large number of variables to a small number of relatively independent “principal components” which still capture a sizeable proportion of variation in the system. By defining a set of 5 “criteria”, CHNRI process effectively reduces a notoriously complex and multi-dimensional task of priority setting, which could be approached through an almost infinite number of “lenses”, into an exercise where the 5 most important (and reasonably independent) criteria for priority setting are clearly defined. They can even be weighted afterwards, in order of their importance to the users.

(ii) “Wisdom of the crowds” – this refers to the process of taking into account the collective opinion of a group of individuals rather than a single expert (or small number of experts) to answer a question, because it has been shown that the average of collective guesses are nearly always closer to the truth than any expert judgement. The pre-requisites for this process to work are: (i) Diversity of opinion (each person should have private information even if it's just an eccentric interpretation of the known facts); (ii) Independence (people's opinions aren't determined by the opinions of those around them); (iii) Decentralization (people are able to specialize and draw on local knowledge); and (iv) Aggregation (some mechanism exists for turning private judgments into a collective decision – in this case, the CHNRI method).

The validation of CHNRI method based on the exercises conducted to date showed: (i) extraordinary stability (correlation coefficients of over 90%) of scores given to same questions by the same experts in different points in time; (ii) almost identical scores of the same question scored by a larger group multiple times (score always falls within +1.7 points on a scale 0-100); and (iii) Monte Carlo simulations in random sub-samples of the larger group of scorers showed that the probability that the outcomes of the exercise could be substantially different if another group of experts conducted the scoring becomes incredibly small as soon as each criterion is scored by more than 17-23 rational persons with some knowledge of the problem; (iv) change of the context of the exercise leads the same group of experts to assign significantly different scores to the same research questions (Rudan I et al., personal communication).

In this paper, we used 16 technical experts to score each criterion. Thus, given the well-defined context for this CHNRI exercise and a set of simple YES/NO questions, it is entirely improbable that any other group of rational individuals with some knowledge of the problem, regardless of their background or selection, would ever reach dramatically different conclusions than our group did.

Although this may seem counter-intuitive to some critics, this is the basic property of the „wisdom of crowds“ phenomenon (for more details please see an excellent book by James Surowiecki: The Wisdom of Crowds: Why the Many Are Smarter Than the Few and How Collective Wisdom Shapes Business, Economies, Societies and Nations), which CHNRI uses as its fundamental principle. Once that each individual gets a right to express judgement that is treated equally as the judgement of any other individual, then the personal biases that those individuals bring into the process tend to cancel and dilute each other regardless who the participants are. What is left is the information based on accumulated knowledge, lifetime experience and common sense of those who took part – which is the result of the CHNRI process.

In comparison to other methods for setting priorities, in “expert panel”-type processes one very loud vote has a potential to heavily bias the process, resulting in shameful inequity and snowballing support for some issues at the expense of the others, a situation which we are observing today. We recently conducted Delphi and CHNRI exercises in parallel to compare them. This happened during the large GAPPS meeting (“Global action plan for prematurity and stillbirth”) sponsored by The Gates Foundation. Nine working groups were defining priorities using Delphi-type process, while three working groups were using CHNRI method. At the end of the conference, the rapporteurs from Delphi groups realized that it is simply not possible to have a discussion on all possible research options and keep in mind all their pros and cons all the time. Eventually, the group leaders ended up forwarding the ideas that they originally brought to the table and gained support for them from the rest of the group. In CHNRI groups, however, a process highlighted pros and cons of many competing ideas. More importantly, after the scoring was conducted, the top priorities were often surprising to the group - because they were frequently the issues which have not been discussed at all, and no-one had expertise in them.

Table w2. Scores assigned to research ideas by the participants in the exercise.

RESEARCH INSTRUMENT

Approaches to reduce smoking in fathers of unborn chidren
during pregnancy

Q.1.1. Would you say the research question is clear and has well defined endpoints?

Victora	Rasmus.	Tomlinson	Singhal	Biloglav	Rudan	Victora	Rasmus.	Tomlinson	Singhal	Biloglav	Rudan	Victora	Rasmus.	Tomlinson	Singhal	Biloglav	Rudan
1	1	1	1	1	1	0.5	1	1	1	1	1	1	1	1	1	1	1
1	1	0.5	0	0.5	0.5	0.5	1	1	1	1	1	1	1	1	1	1	1
1	1	0.5	1	0.5	0.5	0.5	1	1	1	0.5	0.5	1	1	1	1	1	1
1	1	1	1	0	0	1	0.5		1	0.5	0.5	1	1	1	1	1	1
1	1	0	1	0.5	1	0.5	1	1	1	1	1	1	1	1	1	1	1
0.5	1	0	1	0	0	0.5	0.5	1	0	0.5	0.5	1	1	1	1	1	1
0.5	1	0.5	0	0	0	0.5	1	1	0	0.5	0.5	1	1	1	1	1	0.5
0.5	1	1	0	0.5	0.5	0.5	1	1	0	0.5	0.5	1	1	1	1	0.5	1
0.5	1	1	0	1	0.5	0.5	1	1	0	0.5	0	1	1	1	1	0.5	0.5
0.5	1	1	1	0	0	0.5	0.5	1	0.5	0	0	1	1	1	1	1	1
1	1	1	1	1	1	1	1	1	1	1	1	1	1	1	1	1	1
1	1	1	0	0.5	0.5	0.5	0.5	1	0	1	1	1	1	1	1	1	1
0.5	1	0	0	0.5	0.5	0.5	0.5	1	0	0.5	0.5	1	1	1	0	0.5	1
0.5	1	1	1	1	1	0.5	1	1	1	1	1	1	1	1	1	1	0.5
1	1	0	0	1	1	0.5	1	1	0	1	1	1	1	1	1	1	1
1	1	0	0	1	1	0.5	1	0.5	0	1	1	1	1	1	1	1	1
1	1	1	1	0.5	1	0.5	1	1	1	0.5	0.5	1	1	1	1	0.5	1
0.5	1	0.5	0	0	0	0.5	1	0.5	0	0	0.5	1	1	1	1	0.5	0.5
1	0.5	0	0	0.5	0.5	0.5	0.5	0.5	0	0.5	0.5	1	1	1	1	0.5	0.5
1	1	1	0	1	1	0.5	1	1	0	1	1	1	1	1	0	1	1
0.5	1	1	1	1	1	1	1	1	1	1	1	1	1	1	0	1	1
1	1	0	0	1	0.5	1	1	1	0	0.5	0.5	1	1	1	1	1	1
0.5	1	0.5	0	1	1	1	1	0.5	0	1	1	1	1	1	0	1	1
1	1	1	1	1	1	1	1	1	1	0.5	0.5	1	1	1	1	1	1
1	0.5	1	0	1	0.5	0.5	1	0	0	0.5	0.5	1	1	1	1	1	1
0.5	1	1	1	0.5	1	0.5	1	0.5	1	0.5	1	1	1	1	1	1	1
1	1	1	1	0.5	1	1	1	1	1	0.5	1	1	1	1	1	1	1
0.5	1	1	0	1	1	0.5	1	0.5	0	1	1	1	1	1	1	1	1
0.5	1	1	1	0.5	1	0.5	1	1	1	0.5	0.5	1	1	1	1	1	1
0.5	1	0.5	0	1	0.5	0.5	1	1	0	1	0.5	1	1	1	0	1	1
0.5	1	0	0	1	0.5	1	1	1	0	0.5	0.5	1	1	1	0	1	0.5
1	1	1	0	1	1	1	1	0.5	0	1	1	1	1	1	1	1	1
0.5	0.5	1	0	1	1	1	0.5	1	0	1	1	1	1	1	1	1	1
1	1	1	1	1	1	1	1	1	1	1	1	1	1	1	1	1	1
0.5	1	0.5	1	1	1	1	1	1	1	1	1	1	1	1	1		1
1	1	0.5	1	1	1	0.5	1	1	1	1	1	1	1	1	1	1	1
0.5	1	0	1	1	1	0.5	1	0	1	1	0.5	1	1	0.5	1	1	1
0.5	1	1	0	1	1	1	1	1	1	1	1	1	1	1	1	1	1
0.5	1	0	1	1	0.5	1	1	0.5	1	1	0.5	1	1	1	1	1	1
1	1	1	0	0.5	0.5	1	1	1	1	0.5	0.5	1	1	1	1	0.5	0
1	1	0.5	0	0.5	0.5	1	1	0.5	1	0.5	0.5	1	1	1	1	0.5	0.5

1	1	0	0	0.5	0	1	1	0.5	1	0	0	1	1	1	1	1	1
0.5	1	1	1	0.5	0	1	1	1	1	0.5	0	1	1	1	1	0.5	1
0.5	1	1	1	0.5	0.5	1	1	1	1	0.5	0	1	1	1	1	0.5	1
0.5	1	1	0	1	1	1	1	0	1	1	1	1	1	1	1	1	1
0.5	1	1	0	1	0	0.5	1	0	1	0.5	0	1	1	1	1	0.5	1
1	1	1	0	0.5	0.5	0.5	1	1	1	0.5	0.5	1	1	1	1	1	1
0.5	1	0	0	0.5	0.5	1	1	0	1	0.5	0.5	1	1	1	1	0.5	1
0.5	0.5	1	1	0.5	1	1	0.5	0.5	1	0.5	1	1	0.5	1	1	1	1
0.5	1	1	1	1	1	0.5	1	0.5	1	1	1	1	1	1	1	0	0
0.5	1	1	1	1	1	1	1	0.5	1	1	1	1	1	1	1	1	1
1	1	1	0	0.5	1	0.5	0.5	0	0	0.5	0.5	1	1	1	0	1	1
1	1	0.5	1	1	0.5	0.5	1	1	0	1	1	1	1	1	1	1	1
1	1	1	0.5	0.5	0.5	0.5	0.5	1	0.5	0.5	0.5	1	1	1	0.5	0.5	0.5
1	1	1	1	0.5	1	0.5	1	1	1	0.5	1	1	1	1	1	1	1
1	1	1	1	1	1	0.5	1	1	1	1	1	1	1	1	1	1	1
1	1	1	1	0.5	0.5	0.5	1	0.5	0	0.5	1	1	1	1	0	1	1
1	1	1	0	1	1	0.5	1	0.5	1	1	1	1	1	1	1	1	1
1	1	1	0	1	1	0.5	1	0.5	0	1	1	1	1	1	1	1	1
0.5	1	1	0	1	0.5	0.5	1	1	0	1	0.5	1	1	1	1	1	1
1	1	1	1	0.5	0.5	1	1	1	1	0.5	0.5	1	1	1	1	1	1
1	1	1	1	0.5	1	0.5	1	1	1	0.5	1	1	1	1	1	1	1
1	1	1	1	0.5	1	1	1	1	1	0.5	1	1	1	1	1	1	1
1	1	1	1	1	1	1	1	1	1	0.5	0.5	1	1	1	1	1	1
0.5		1	1	0.5	0.5	0.5		0.5	1	0.5	0.5	1		1	1	1	0.5
0.5	1	0.5	0	1	1	0.5	1	0.5	0	0.5	0.5	1	1	1	1	1	1
1		1	1	0.5	1	1		0.5	1	0.5	0.5	1		0.5	1	1	1
1	1	1	0	1	1	0.5	0.5	1	0	1	1	1	0.5	0.5	1	1	1
1	1	1	1		0.5	0.5	1	0.5	1		0.5	1	1	0.5	1	1	1
0.5	1	0.5	1	1	1	0.5	1	0	1	1	0.5	1	1	1	1	1	1
1	1	0.5	1	0.5	0.5	1	1	0.5	1	0.5	0.5	1	1	1	1	1	1
0.5	0.5	0	0	0.5	0.5	0.5	0.5	0.5	0	0.5	0.5	1	0.5	1	1	0.5	0.5
0.5	1	1	1	1	1	1	1	0	1	0	0	1	1	0	1	0	0
0.5	1	1	1	1	1	0.5	1	0	1	0.5	0.5	1	1	0.5	1	0	1
1	1	0.5	1	0.5	0	1	1	0.5	1	0.5	0.5	1	1	0.5	1	0.5	1
1	1	0	0	0.5	1	0.5	1	0	1	0.5	0.5	1	1	0.5	1	1	1
0.5		0.5	0	1	1	0.5		0	1	0	0	1		0	1	0	0.5
0.5		1	1	1	1	0.5		0	1	0	0	1		0	0	0	1
0.5	1	1	1	0.5	0.5	1	1	0.5	1	0.5	1	1	1	0	1	0.5	1
0.5	1	1	0	1	1	1	0.5	0	0	1	0.5	1	1	0.5	1	0.5	1
1		1	0	1	1	1		0.5	0	0.5	0	1		0	1	0.5	1
1		1	0	1	1	1		0.5	0	0.5	0.5	1		0.5	1	1	1

Q.2.1. Based on the best existing evidence and knowledge, would the intervention(s) which would eventually benefit from the proposed research be efficacious in reducing diarrhoea-related mortality?																	
Sachdev	Edmond	Rasmus.	Kramer	Mori	Bhutta	Sachdev	Edmond	Rasmus.	Kramer	Mori	Bhutta	Sachdev	Edmond	Rasmus.	Kramer	Mori	Bhutta
0	0.5	1	1	0.5	1	0	0.5	1	1	0.5	1	1	0.5	1	0.5	1	1
1	0	1	1	0.5	1	1	0	1	1	0.5	1	1	0	1	0.5	1	1
1	0.5	1	1	0.5	0	1	0.5	1	1	0.5	0	1	0.5	1	1	0	1
0	0	1	1	0.5	0	0	0	1	1	0.5	0	0	0	0.5	0.5	1	0
1	0	1	0	0	1	1	0	1	0	1	1	1	0	1	0	0	1
1	0	1	0	0	1	1	0	1	0	1	0	0	0	1	0	0	0.5
1	0	1	0	0	0.5	1	0	0.5	0	0	0	0.5	0	0.5	0	0	0.5
1	1	1	1	1	1	1	1	0.5	1	0	1	0.5	1	0.5	0.5	1	1
1	1	1	1	1	0	1	1	0.5	1	0	0	0.5	1	1	0.5	0	1
0.5	1	0.5	0.5	1	0		1	0.5	0.5	0	0	0	1	0.5	0.5	0	1
0	0	1	0	1	1	0	0	1	0	1	0	1	0	1	0	1	1
1	1	1	0	0	0	1	1	1	0	1	0	0.5	1	0.5	0	1	0
0	0	1	0	0	0	0	0	0.5	0	1	0	0	0	0.5	0	1	1
0	0	1	0	1	0.5	0	0	0.5	0	0	0.5	0.5	0	0.5	0	1	1
0.5	1	1	0.5	1	0	0.5	1	1	0.5	0	0	0	1	0.5	0.5	0	0
0.5	1	1	0.5	1	0	0.5	1	1	0.5	0	0	0	1	0.5	0.5	0	0
0	0	1	0	1	1	0	0	0.5	0	0	1	0.5	0	1	0	0	1
0	1	1	0	0	1	0	1	0.5	0	1	0	0.5	1	1	0	0	1
0	1	1	0.5	0	0	0	1	0.5	0.5	1	0	1	1	1	0.5	0	1
1	1	1	0	1	0.5	1	1	1	0	0	0	0.5	1	0.5	0	1	0.5
0	1	1	0	1	0	0	1	1	0	0	0	0.5	1	0.5	0	0	0
0.5	1	1	1	1	0.5	0.5	1	1	1	0	0	1	1	0.5	0.5	0	0
1	1	1	1	0	1	1	1	1	1	1	0	0.5	1	0.5	0.5	0	1
1	1	1	1	0	1	1	1	0.5	1	1	1	1	1	0.5	1	1	1
1	1	1	0	0	1	1	1	1	0	1	1	0.5	1	0.5	0	0	1
1	1	1	0.5	0	1	1	1	1	0.5	1	1	0.5	1	0.5	0	1	1
0.5	1	1	0	0	1	0.5	1	0.5	0	1	0	0.5	1	0.5	0	0	0
1	1	1	0.5	0	1	1	1	0.5	0.5	1	1	1	1	0.5	0.5	1	1
1	0	1	0	0	1	1	0	0.5	0	1	1	0.5	0	0.5	0	1	1
0.5	1	1	0	0	1	0.5	1	1	0	1	1	0.5	1	0.5	0	1	1
1	0	1	0	0	1	1	0	1	0	1	1	1	0	1	0	0	1
1	1	1	1	1	1	1	1	1	1	1	1	1	1	1	0.5	1	1
1	1	1	1	1	1	1	1	1	1	1	1	1	1	1	1	1	1
0.5	1	1	0	0	1	0.5	1	1	0	1	0	0.5	1	0.5	0	1	0.5
1	1	1	1	0	1	1	1	0.5	1	1	1	0.5	1		1	0	1
0.5	1	1	0	1	1	0.5	1	1	0	1	0	0	1	0.5	0	1	0.5
1	1	1	0.5	0	1	1	1	1	0.5	1	1	1	1	1	0.5	1	1
1	1	1	0	0	1	1	1	1	0	1	1	1	1	1	0	1	1
0.5	1	1	0	0	1	0.5	1	0.5	0	1	0.5	0.5	1	0.5	0	0	1
0.5	1	1	0	1	1	0	1	1	0	1	1	0.5	1	1	0	1	1
0	1	1	0	1	1	0	1	1	0	1	0	0	1	1	0	0	0.5

0.5	0	1	0	1	0	0.5	0	0.5	0	0	0	0.5	0	0.5	0	0	0
0.5	0	1	0	1	0	0.5	0	1	0	0	0	0	0		0	0	0
0.5	0	1	0	1	0	0.5	0	1	0	0	0	0	0		0	0	0
0.5	0	1	0.5	1	1	0	0	1	0.5	0	0.5	0	0	0.5	0.5	0	1
1	1	1	0	1	1	1	1	0.5	0	0	0	0	1	0.5	0	0	0
0.5	1	1	0	1	1	0.5	1	1	0	1	1	1	1	1	0	1	1
0.5	1	1	0	1	1	0.5	1	0.5	0	1	1	0	1	0.5	0	1	1
0.5	0.5	1	0	1	0	0.5	0.5	1	0	1	0	0.5	0.5	0.5	0	1	1
	1	1	1	1	1		1	1	1	1	1	0	1	0.5	1	1	1
	1	1	0.5	1	1		1	1	0.5	1	1	0	1	0.5	0.5	1	1
1	0.5	1	1	1	1	1	0.5	1	1	1	0.5	0	0.5	0.5	1	1	1
1	1	1	1	1	1	1	1	1	1	1	1	1	1	1	1	1	1
	0	1	0.5	1	1		0	1	0.5	1	0.5	1	0	1	0.5	1	1
	0	1	0.5	0	1		0	1	0.5	0	1	0	0	1	0.5	0	1
0.5	1	1	0.5	1	0.5	0.5	1	1	0.5	1	0	1	1	1	0	1	1
0.5	0	1	1	1		0.5	0	1	1	1		0.5	0		0.5	1	
0.5	1	1	0	1	0	0.5	1	1	0	1	0	0	1	1	0	1	0
0	1	1	0	1	0	0	1	1	0	1	0	0.5	1	1	0	1	0
0	1	1	0	1	0.5	0	1	1	0	1	0	0	1	1	0	1	0
1	1	1	1	1	1	1	1	1	1	1	1	1	1	1	0.5	1	1
	0	1	1	1	1		0	1	1	1	0	0	0		0.5	1	0.5
0.5	1	1	0	0	1	0.5	1	1	0	0	1	0.5	1		0	0	1
1	1	1	0.5	1	1	1	1	1	0.5	1	1	1	1		0.5	1	1
0	1	1	0.5	1	0	0	1	1	0.5	1	0	1	1		0.5	1	0
1	1	1	1	0	1	1	1	1	1	0	1	0	1	1	0.5	0	1
1	1		1	1	1	1	1		1	1	1	0	1		0.5	1	1
	1	1	0.5	1	1		1	1	0.5	1	1	0	1	1	0.5	1	1
0.5	1	1	0.5	1	1	0.5	1	1	0.5	1	1	1	1	0.5	0.5	1	1
1	1	1	0.5	1	0	1	1	1	0.5	1	0	0	1	1	0	1	0.5
0.5	0	1	0	1	1	0.5	0	1	0	1	1	1	0	1	0	1	1
	1	1	0	1	0.5		1	1	0	1	0	0	1	1	0	1	0
	1	1	0	1	0		1	1	0	0	0	0	1	0.5	0	0	0
0.5	0	1	0.5	1	0	0	0	1	0.5	1	0	0.5	0	1	0.5	1	0
1	1	1	1	1	1	1	1	1	1	1	1	1	1		0.5	1	0.5
0.5	1	1	0	1	1	0.5	1	1	0	1	1	0.5	1		0	1	1
	1		0	1	1		1		0	1	0	0	1		0	1	0
	1		0	1	0		1		0	1	0	0	1		0	1	0
0.5	1	1	0.5	1	0	0	1	1	0.5	1	0	0.5	1		0	1	0
0.5	0	1	0	1	1	0	0	1	0	0	0	0	0	0.5	0	0	0
	1	1	0	1	1		1	0.5	0	1	1	0	1		0	1	1
0.5	0	1	0	1	1	0	0	1	0	1	1	0	0		0	1	1

Q.3.1. Taking into account (i) the infrastructure and resources required to deliver effective interventions (e.g. human resources, health facilities, communication and transport infrastructure), and (ii) the need for change in demand, beliefs and attitudes of users, would you say that the endpoints of the research would be deliverable (or the findings of this research would improve deliverability of other interventions)?

Williams	Manandhar	Singhal	Bhandari	Iyengar	Mori	Williams	Manandhar	Singhal	Bhandari	Iyengar	Mori	Williams	Manandhar	Singhal	Bhandari	Iyengar	Mori
1	1	0	0	1	0.5	1	0	1	0	1	0.5	1	0	1	0	1	0.5
1	1	0	0	1	0.5	1	1	1	0	1	0.5	1	1	1	0	1	0.5
0	1	0	0	0	0.5	0	0	1	0	1	0.5	0	0	1	0	0.5	0.5
1	1	1	0	1	0.5	1	1	1	0	0.5	0.5	1	1	1	0	0.5	0.5
1	1	0	1	1	1	1	1	0	1	1	1	1	1	0	1	1	1
0	1	0	0	0	0	0	1	0	0	0	0	0	1	0	0	1	0
1	1	0	0	0	0.5	1	1	0	0	0	0.5	1	0	0	0	0.5	0.5
1	1	1	0		1	1	1	1	0	0.5	0	1	1	1	0	0	0
1	1	1	0	0.5	1	1	1	1	0	1	0	1	1	1	0	0.5	0
1	1	1	0.5		1	1	1	1	0.5		0	1	1	1	0.5		0
1	1	0	0	1	0.5	1	1	1	0	1	0.5	1	1	1	0	1	0.5
1	1	0	0.5	0	1	1	0	0	0.5	0	0	1	0	0	0.5	0.5	0
0	1	0	1	1	1	0	1	0	1	1	1	0	1	0	1	1	0
1	1	1	0	1	1	1	1	1	0	1	1	1	0	1	0	0	0
1	1	0	0	0.5	1	1	1	1	0	0	1	1	0	1	0	1	1
1	1	1	0	1	1	1	1	1	0	0	1	1	1	0	0	1	1
0	1	0	0	0	1	1	0	0	0	0	0	1	0	0	0	0	0
1	1	0	0	1	1	0	0	0	0	1	0	0	0	0	0	0	0
0	0	0	0	0	1	0	0	0	0	0	0	0	0	0	0	0	0
1	1	0	0.5	1	1	1		0	0.5	1	1	1	0	0	0.5	1	0
0	1	1	1	1	1	1	1	1	1	1	0	1	1	1	1	0.5	0
1	1	0	0	1	0.5	1	1	0	0	1	0.5	1	1	0	0	0	0.5
1	1	1	1	1	1	0.5	1	1	1	1	1	0.5	1	1	1	1	1
1	1	1	0.5	1	1	1	1	1	1	0.5	1	1	1	1	1	0	1
0	1	1	0.5	1	1	1	1	1	1	1	1	1	1	1	1	1	1
1	1	1	1	1	1	1	1	1	1	1	0	1	1	1	1	0.5	0
0	1	0	1	1	0	0	1	0	1	1	0	1	1	0	1	0.5	0
1	1	0	1	1	1	1	1	0	1	0.5	1	1	1	0	1	1	0
1	1	1	0.5	0.5	1	1	1	1	1	1	1	1	1	1	0.5	0.5	0
1	1	0	1	1	1	0	1	0	1	1	1	0.5	1	0	1	0.5	0
1	1	0	0.5	1	1	1	1	0	0.5	1	1	1	1	0	0.5	0.5	0
0	1	1	0.5	1	1	0.5	1	1	0.5	1	1	0.5	1	1	0.5	1	1
1	1	1	1	1	1	0.5	1	1	1	1	1	0.5	1	1	1	1	1
0	1	0	0.5	0	1	0	1	0	0.5	1	1	1	1	0	0.5	0.5	0
1	1	1	0.5	0.5	1	0.5	1	1	1	0	1	0.5	1	1	1	0	1
1	1	0.5	0.5	1	1	0.5	1	1	1		1	0.5	1	1	0		1
0	1	1	1	1	1	0	1	1	1	1	1	0	1	1	0.5	0.5	1
1	1	0	0.5	1	1	1	1	0	1	1	1	1	1	0	0.5	1	1
1		0	1	1	1	0.5	1	0	1	1	1	0.5	1	0	0.5	1	1
0	1	0	1	0	1	0	1	0	1	0	1	0	1	0	0	0.5	1
0	0	0	1	1	1	0	0	0	1	0	1	0	0	0	0.5	0.5	1

0	1	0	0	1	1	0	1	0	0	1	0	0	1	0	0	1	0
1	1	0	0.5	1	1	1	1	0	0.5	0.5	0	1	1	0	0.5	0.5	0
1	1	0	0.5	1	1	1	1	0	0.5	0.5	0	1	1	0	0.5	1	0
1	1	0	0.5		1	1	1	0	0.5		1	1	1	0	0.5		0
1	1	0	0.5	0.5	1	1	1	0	1	1	0	1	1	0	0.5	1	0
0		1	0.5	0	0	0		0.5	0.5	0	0	0		0.5	0.5	0	0
		0	0.5	0.5	0			0	0.5	0	0			0	0.5	1	0
	1	0	0	0	1		1	0	0	1	1			0	0	1	0
1	1	1	1	1	1	1	1	1	1	1	1	1	1	1	1	1	1
1	1	1	0	1	1	1	1	1	0	1	1	0.5	1	0	0	1	1
1	1	0	0	0	1	1	1	0	0	0	1	1	1	0	0	1	1
1	1	1	1	1	1	1	1	1	1	1	1	1	1	0.5	1	1	1
0	1	0.5	1		1	0.5	1	0.5	1		1	0.5		0.5	1		1
0	1	1	1	0	1	0	1	1	1	0	1	0	1	1	1	0	1
1	1	1	0.5	1	1	1	1	1	1	1	1	1	1	1	0.5	1	1
1	1	1	0	0.5	1	1	1	0.5	0	1	0	1		0	0	1	0
1	1	0	0	1	1	1	1	0	0	1	1	1	1	0	0	1	1
1	1	0	0	0	1	1	1	1	0	1	1		1	0	0	0	0
1	1	0	0	0	1	1	1	1	0	1	1	1	0.5	0	0	0	1
1	1	0	0.5	1	1	1	1	1	1	1	1	1	1	0	0.5	1	0
0	1	1	0	1	1	0.5	1	1	1	1	1	0	1	1	0	1	1
1	1	1	1	1	1	1	1	1	1	0.5	1	1	1	1	0.5	1	1
1	1	0	1	1	1	1	1	1	1	1	1	1	1	0	0.5	1	0
1	0.5	0	0	1	1	1	0.5	0	0	0	1	1		0	0	0	0
1	1	1	1	1	1	1	1	1	1	1	1	0.5	1	0	0.5	0	1
0	1	1	0	0	1	0	1	1	0	0	0	0	1	1	0	0	0
1	1	0	0	1	1	1	1	0	0	1	1	1	0.5	0	0	1	0
0	1	1	0	0	1	0	1	1	0	1	1	0	1	1	0	1	1
1	1	0	1	1	1		1	1	1	0	1		1	0	0.5	0	1
1	1	0	0.5	0.5	1	1		0	0.5	0	1	1		0	0.5	0.5	0
0		0	0	0	1	0		0	0	0	1	0		0	0	0	0
0	1	0	0	1	1	0	1	1	0		0	0		0	0	0.5	0
0	1	0	0	1	1	0	1	1	0		0	0		0	0	0	0
1	1	1	0.5	1	1	1	1	1	0.5	1	1	1	1	1	0.5	1	0
1	1	1	0.5	1	1	1	1	1	0.5	0	1	1	1	1	0.5	0.5	1
0	1	0	0	0	0	0		0	0	0	0	0		0	0	0	0
0	1	0	0	0	0	0		0	0	0	0	0		0	0	0	0
0	1	0	0.5	1	1	0	1	0	1		1	0		0	0.5		0
0	1	0	0	0	1	0	1	0	0	0	0	0		0	0	0	0
0	1	0	1	1	1	1	1	0	1	0.5	0	1		0	1	1	0
1	1	0	0	1	1	1	1	0	0	0.5	1	1	1	0	0	1	0

Williams Manandhar

0	1	1	0.5	0	0	0	0	1	0	0.5	0	0	0	0	0	0	0.5	0	0	0
1	1	1	1	0	0	0	0	1	0	1	0	0	0	0	0	0	1	0	0	0
1	1	1	1	0	0	0	0	1	0	1	0	0	0	0	0	0	1	0	0	0
1	1	1	0.5	1	0	0.5	1	1	0	0.5	0	0	0	0	0	0	0.5	0	0	0
1	1	0	1	0	1	0.5	0	1	0	1	0	0	0	0	0	1	1	0	0	0
0	1	0	1	0	0	1	0	1	0	1	0	0	0.5	0	0	0	1	0	0	0
0	1	1	1	0	0	1	0	0	0	1	0	0	0	0	0	0	1	0	0	0
1	1	1	0	0	0	0	0	0	0	0	0	0	0	0	0	0	0	0	0	0
1	1	0	1	1	1	1	1	1	1	1	1	0	1	1	0	0	1	1	0	0
1	1	0	1	1	0.5	0.5	1	1	1	1	0.5	0	0	1	1	0	1	0	0	0
1	1	0	1	1	0	1	1	1	1	1	1	0	1	0	0	0	1	1	0	0
1	1	0	1	1	1	1	1	1	0	1	0.5	0	0.5	1	1	1	1	0	0	0
1	1	0	0	1	1	0	0	1	1	0	0	0	0	0	0	0	0	0	0	0
1	1	0	0	0.5	0	1	0	1	1	0	0	0	0	0	0	0	0	0	0	0
1	1	1	1	0.5	1	0.5	0	1	0	1	0	0	0	0	1	0	1	0	0	0
1	1	1	0	1	0		1	1	0	0	1	0		0	0	0	0	0.5	0	
1	1	0	0.5	0	0	0	0	1	1	0.5	0	0	0	0	0	0	0.5	0	0	0
0	1	0	1	0	0	0	0	0	0	1	0	0	0	0	0	0	1	0	0	0
1	1	0	0.5	0	0	0	0	0	0	0.5	0	0	0	0	0	0	0.5	0	0	0
1	1	0	1	1	1	1	0	1	1	1	0.5	0	1	0	0	0	1	0	0	0
1	1	1	1	1	0.5	1	0	1	0	1	0.5	0	0	0	0	0	1	0	0	0
1	1	1	1	0	0.5	1	0	1	0	1	0	0	0	0	0	0	1	0	0	0
1	1	0	1	1	0.5	1	0	1	1	1	1	0	1	0	0	0	1	0.5	0	0.5
1	1	1	1	1	0	0	0	0	0	1	0.5	0	0	0	0	0	1	0	0	0
1	1	1	1	1	1	1	1	1	0	1	1	0	1	0	1	0	1	1	0	1
1	1	0	0.5	1	1	1	0	1	1	0.5	1	0	1	0	0	0	0.5	1	0	0
1	1	0	0.5	0.5	0	1	1	1	0	0.5	0	0	0	1	0	0	0.5	0	0	0
1	1	0	1	0.5	0	1	0	1	0	1	0	0	0	0	0	0	1	0	0	0
1	1	1	1	0.5	0.5	0	0	1	0	1	0	0	0	0	0	0	1	0	0	0
1	1	1	0.5	0	1	1	0	1	0	0.5	0	0	1	0	0	0	0.5	0	0	0
0	1	0	1	0	0	0	0	0	0	1	0	0	0	0	0	0	1	0	0	0
1	1	1	1	0	0	0	0	1	0	1	0	0	0	0	0	0	1	0	0	0
0	1	1	1	0.5	0	0	0	1	0	1	0	0	0	0	0	0	1	0	0	0
1	1	0	1	1	1	1	0	1	1	1	0.5	0	1	0	1	0	1	0	0	0
1	1	0	1	0	1	1	0	1	1	1	0	0	0	0	1	0	1	0	0	0
1	1	0	1	0	0	0.5	1	1	0	1	0	0	0	0	0	0	1	0	0	0
1	1	0	0.5	0	0	0	0	1	0	0.5	0	0	0	0	0	0	0.5	0	0	0
0	1	0	1	0.5	0.5	0	0	1	0	1	0	0	0	0	1	0	1	0	0	0
0	1	0	0.5	0	0	0	0	1	1	0.5	0	0	0	0	1	0	0.5	0	0	0
1	1	1	1	0	0	1	0	1	0	1	0	0	0	0	1	0	1	0	0	0
1	1	1	0	0	0	1	0	1	0	0	0	0	0	0	0	0	0	0	0	0

Q.5.1. In given context, would you say that the present distribution of disease burden primarily addressed by the proposed research affects mainly the underprivileged in the population?																	
Victora	Lawn	Tomlinson	Biloglav	Iyengar	Rudan	Victora	Lawn	Tomlinson	Biloglav	Iyengar	Rudan	Victora	Lawn	Tomlinson	Biloglav	Iyengar	Rudan
1	1	1	1	1	1	1	1	1	1	0.5	1	0.5	1	1	1	1	1
1	1	1	1	1	1	1	1	1	1	1	1	0.5	1	1	1	1	1
1	1	1	1	1	1	1	1	1	1	0	1	0.5	1	1	1	1	1
1	1	0.5	0.5	1	0	1	0.5	0.5	1	1	1	0.5	0.5	0	1	1	1
1	1	1	0.5	1	0	1	1	1	1	1	1	0.5	1	1	1	1	1
1	0.5	0	0.5	0	0	1	0.5	1	1	0	1	0.5	0.5	1	0.5	1	0
1	1	1	0.5	1	1	1	0.5	1	0.5	0	1	0.5	0.5	1	0.5	1	0
1	1	1	1	1	1	1	1	1	0.5	1	1	0.5	1	1	0.5	0	0
1	1	1	1	0	1	1	0.5	1	0.5	1	1	0.5	0.5	1	0.5	0	0
1	1	0.5	0		0	1	0	1	1		1	0.5	0	1	0.5		0
1	1	1	1	1	1	1	0.5	1	1	1	1	0.5	0.5	1	1	1	1
1	1	0.5	1	1	0	1	0.5	1	1	1	1	0.5	0.5	1	1	1	1
1	1	0	1	1	1	1	1	0	0.5	1	1	0.5	1	0	1	0	1
1	1	0.5	1	1	1	1	0.5	0.5	1	1	1	0.5	0.5	0	1	0	1
1	1	0	1	1	1	1	0.5	0.5	1	1	1	0.5	0.5	0	1	1	1
1	1	0.5	1	1	1	1	0.5	0.5	1	1	1	0.5	0.5	0.5	1	1	1
1	1	1	0.5	1	1	1	0.5	1	1	0	1	0.5	0.5	1	0.5	1	1
1	1	0.5	0.5	1	1	1	0	0.5	1	1	1	0.5	0	0.5	0	0	0
1	1	0	0.5	0	1	1	0	0	1	0	1	0.5	0	0	0	1	0
1	1	1	1	1	1	1	0	1	1	1	1	0.5	0.5	1	1	1	1
1	1	1	1	1	0	1	0.5	1	1	1	1	0.5	0.5	1	1	1	1
1	1	0.5	0.5	0	0.5	1	1	1	1	0	1	0.5	1	1	1	1	1
1	1	0	1	1	1	1	0.5	0.5	1	1	1	0.5	1	1	1	1	1
1	1	0.5	1	1	1	1	1	1	1	1	1	0.5	1	1	1	0	1
1	1	0.5	1	1	1	1	0.5	1	1	1	1	0.5	1	1	1	1	1
1	1	1	1	1	1	1	1	1	1	1	1	0.5	1	1	1	1	1
1	1	1	1	1	0	1	0.5	1	1	1	1	0.5	0.5	1	1	0	1
1	1	1	1	1	1	1	1	1	1	1	1	0.5	1	1	1	1	1
1	1	1	1	0.5	1	1	1	1	1	0.5	1	0.5	1	1	1	1	1
1	1	1	1	1	1	1	1	1	1	1	1	0.5	1	1	0.5	1	1
1	1	1	0.5	0	0	1	1	1	1	0	1	0.5	1	1	1	1	1
1	1	0	1	0.5	1	1	1	0.5	0.5	1	0.5	0.5	1	0.5	1	1	1
1	1	0.5	0	1	0	1	0.5	0.5	0.5	1	0.5	0.5	1	1	1	1	1
1	1	0	0	0	0	1	0.5	0.5	0.5	1	0.5	0.5	1	0	1	0	1
1	1	0.5	1	0.5	1	1	1	0.5	1	1	1	0.5	1	0.5	1	0	1
1	1	1	0	0	0	1	0.5	1	1	0	1	0.5	1	1	1	1	1
1	1	1	1	1	1	1	1	1	0.5	1	1	0.5	1	1	1	1	1
1	1	1	1	1	1	1	1	1	1	1	1	0.5	1	1	1	1	0
1	1	1	1	1	1	1	1	1	1	1	1	0.5	1	1	1	1	1
1	1	1	0.5	0	0.5	1	0.5	1	0	0.5	0	0.5	1	1	1	0	0.5
1	1	1	0.5	0	0.5	1	1	1	0	0.5	0	0.5	1	1	1	0.5	0.5

1	0.5	0.5	0.5	1	0	1	0	0.5	0.5	0.5	0.5	0.5	0.5	1	0.5	0	0
1	1	0.5	0.5	1	1	1	1	0.5	0.5	1	0	0.5	1	1	0.5	1	0
1	1	0.5	0.5	0.5	1	1	1	0.5	0.5	0.5	0.5	0.5	1	1	0.5	1	0.5
1	1	0.5	0		0.5	1	0	1	1		0	0.5	0.5	1	1		0.5
1	1	0.5	1	1	0.5	1	1	0.5	0.5	1	0	0.5	1	1	1	1	1
1	1	1	1	0	1	1	0.5	1	0.5	0	0.5	0.5	0.5	1	1	1	1
1	1	0	0.5	1	1	1	0.5	0	0.5	0	1	0.5	1	0.5	0.5	1	1
1	1	0.5	1	1	0.5	1	1	0.5	1	1	0.5	0.5	1	1	1	0	1
1	1	1	0	0	1	1	1	1	1	0	1	0.5	1	1	1	1	1
1	1	1	1	1	1	1	1	1	1	1	1	0.5	1	1	1	1	1
1	1	1	1	0	1	1	0.5	1	1	0	0	0.5	1	1	1	0	1
1	1	0	0	1	0	1	0.5	0.5	1	1	1	0.5	1	0.5	1	1	1
1	1	1	0.5		0.5	1	1	1	0.5		0.5	0.5	1	1	1		1
1	1	1	0	1	0	1	0.5	1	0	1	0	0.5	1	1	0.5	0	1
1	1	1	1	1	1	1	0	1	0.5	1	0.5	0.5	0	1	1	1	1
1	1	0.5	0.5	1	0.5	1	1	0.5	0.5	1	0.5	0.5	1	0.5	1	1	1
1	1	0	0.5	1	0.5	1		0	1	1	0.5	0.5		0.5	1	1	1
1	1	0	0.5	0	0.5	1		0	1	0	1	0.5		0	1	0	1
1	1	0	0.5	1	1	1		0	1	1	1	0.5		0.5	1	1	1
1	1	1	1	1	1	1		1	1	1	1	0.5		1	1	1	1
1	1	1	1	1	1	1		1	1	1	1	0.5		1	1	0	1
1	1	1	1	1	1	1	1	1	0.5	1	0.5	0.5	1	1	1	1	1
1	1	0.5	1	1	1	1	1	1	0.5	1	0	0.5	1	1	1	1	1
1	1	0	1	0	1	1	0	0.5	0.5	0	0.5	0.5	0.5	1	1	1	1
1	1	0	1	0	1	1	1	0	0.5	1	0.5	0.5	1	1	1	1	1
1	1	0	1	1	1	1	0.5	0	1	0	1	0.5	1	0.5	1	1	1
1	1	0	0.5	0	1	1	0.5	0	0	0	0	0.5	1	0.5	1	1	1
1	1	0.5	0.5	1	0.5	1	0.5	0.5	1	0	1	0.5	1	0.5	1	1	1
1	1	1	1	1	1	1	0.5	1	0.5	0	0.5	0.5	1	1	1	1	1
1	1	1	1	1	1	1	0.5	1	0.5	0	0.5	0.5	1	1	1	1	1
1	1	0	0.5	0	0.5	1	0.5	0	0.5	0	0.5	0.5	0.5	0	0.5	0	0.5
1	1	0	1	0.5	0.5	1	0.5	0	0	0	0	0.5	0.5	0	0	1	0
1	1	1	1	0	1	1	0	1	0	0	0	0.5	0.5	1	0.5	0	1
1	1	1	1	1	1	1	0.5	1	0.5	1	0.5	0.5	1	1	1	1	1
1	1	0.5	1	1	1	1	1	0.5	0.5	0	0	0.5	1	0.5	0.5	0	0.5
1	1	0	0.5	0.5	0.5	1	0	0	0	0	0	0.5	0	1	0	1	0
1	1	0	1	1	1	1	0	0	0	0	0	0.5	0	0.5	0	0	0
1	1	0	0.5	0	0.5	1	0.5	0	0	0	0	0.5	0.5	0.5	0	0	0
1	1	0	0.5	1	0.5	1	0	0	0	0	0	0.5	0	0	0	0	0
1	1	0.5	0.5	1	0.5	1	1	0.5	0.5	1	0	0.5	1	0	0.5	1	0
1	1	0.5	0.5	1	0.5	1	0.5	0	0.5	0	0	0.5	1	0	0.5	1	0

Table w3. The final list with CHNRI research priority scores (RPS) and average expert agreement (AEA) for all proposed research ideas.

RANK	RESEARCH QUESTION	N	ANSWER	EFFECTIV	DELIVER	BURDEN	EQUITY	TOTAL (unwgh)		

41	Population studies to determine mortality associated with preterm birth at gestations 24-28 weeks, 28-32 weeks and 32-36 weeks using verbal autopsy, and the time of death (first day, first week, first month, post-neonatal period)									
